# Cruel Visions: Reflections on Artists and Atrocities

**DOI:** 10.1080/14434318.2020.1764226

**Published:** 2020-08-05

**Authors:** Joanna Bourke

War hurts. When hurled into armed conflict, the artist faces the formidable task of balancing the poetics of revelation against the aesthetics of destruction. Uniquely, artists who are dispatched into the cauldron of combat from regions of the world that are largely free from war are forced to recognise that the body—*their* body—is no longer a subject ‘good to think’ but an object that is ‘necessary to be’.^1^

In this article, I explore some of the artistic difficulties facing officially appointed war artists seeking to visually represent atrocities such as rape. There is a sophisticated literature reflecting on the art of trauma. Artists such as Martha Rosler, Alfredo Jaar, Sophie Ristelhuber, Simon Norfolk, James Bridle, Gervasio Sánchez, and Gustavo Germano have created powerful artistic responses to traumas such as combat, mass killings, ‘disappearances’, and rape. *Official* war artists, however, are required to adopt a different aesthetic. Moralists repeatedly warn against conflating personal trauma with secondary witnessing, but I maintain that we need to take seriously the idea that the sights, sounds, smells, tastes, and touch of the wounded body can destroy worlds beyond the immediate victims. This article is an attempt to accentuate the role of embodiment in artistic constructions of the meaning of wartime atrocity. The term ‘embodiment’ draws inspiration from theorists who argue that people *think* via sensorimotor experiences: our minds are embodied. As Raymond W. Gibbs has evocatively stated, ‘cognition is what happens when the body meets the world’.^2^ This article also introduces the idea that empathy emerges as a capacity of imaginative embodiment.

This is a departure from much of the literature. Historians of official war artists and the majority of these artists (of either sex) tend to share a masculinist ethos that sidelines, ignores, or even denies the artist’s body. At best, the fleshy physicality of the artist is viewed as nothing more than an *instrument* of imaginative agency. Attention tends to be focused on the artist as a rational, aesthetically disembodied human subject. The artist’s body is regarded as irrelevant not only to the production of images themselves, but also to ethical decision-making. In contrast, I am interested in embodied approaches to the construction of artistic meaning and empathy in war. Basic bodily movements, such as agitated brush marks, broad strokes, thick scrapings of pigment, and frenzied jabs, provide forms of knowledge—they help to create and even connect the ‘poetics of revelation’ and ‘the aesthetics of destruction’.

Militarily embedded artists, whether commissioned by state authorities, media conglomerates, or other institutional agencies (such as national galleries), are not usually placed in contexts in which they become perpetrators of violence. While in moments of crisis some artists do engage directly in battle, they usually only encounter extreme cruelty and murder through acts of sense perception. The artist at the scene of war’s carnage is unable to stand outside the spectacle of atrocity. Witnessing the suffering intrinsic to battle cannot be isolated from all other aspects of the artist’s life. In other words, from the moment of sense perception, atrocities are interpreted through the lens of the artist’s entire life story, including his or her infant attachment relations, adult interpersonal bonds, fleshy vulnerabilities, and cognitive frames, not to mention the artist’s exchanges with people in pain and their tormentors. This process is fundamentally embodied. As Maurice Merleau-Ponty insisted, ‘to perceive is to render oneself present to something through the body’.^3^ His message is: people don’t *have* bodies, they *are* bodies.^4^ By this, he does not mean we are nothing more (or less) than physiological flesh; rather, we are an indivisible mix of physiology (neurological pulses, autonomic arousal, cardiovascular responses, and sensorimotor actions, for example), affect (fear, hatred, happiness), the unconscious (projection, sublimation), and cognition (including ideology). This complex, embodied artist is intrinsically interconnected with the Other, including that other person’s trauma. Artistic witnessing, therefore, requires a repudiation of the Cartesian distinction between the body and mind, as well as a radical rethink about the inequalities that mark people’s lives—including the inequality that distinguishes the life of the artist from that of the victim of atrocity.

The representation of war atrocities in art has generated a large and productive literature in the past few decades. Typical questions include: is it even possible to capture the horrors of warfare—let alone atrocities—in paint or pencil, crayon or celluloid, dyes or digital technologies? Hundreds of scholars have responded to the powerful reflections of scholars such as Susan Sontag in her *Regarding the Pain of Others* and *On Photography*.^5^ Questions have also been raised about the aesthetics of atrocity.^6^ Is there a risk that visually representing a brutal act will reproduce its violent obscenity? Might viewers become accustomed to barbarian ways or, worse, end up celebrating death and openly fetishising courage, gallantry, and honour? And isn’t there a risk of repeating the great lie of war: that suffering is redemptive?^7^

This article will focus on a small number of commissioned war artists, including Linda Kitson, John Keane, and David Rowlands, but particular attention will be paid to Peter Howson’s art from the conflict in Bosnia. Howson is a Scottish artist who, at the age of thirty-five, was chosen by the Imperial War Museum (IWM) and *The Times* newspaper to serve as the official war artist in Bosnia. He was embedded in the British Army contingent attached to the UN Peacekeeping Force. From the start, he promised that he would not come back with ‘sketches of still lives but would get as near to the fighting as possible’.^8^ Howson’s art from the conflict in Bosnia is an intricate and inimitable example of how an artist—and, specifically, a *male* artist—can reflect on the traumas of rape. Howson actively reflects on questions of empathy and engagement. He self-consciously plays on notions of ‘home’ and the tensions intrinsic to maintaining a masculinist persona while immersing himself in gendered violence against women. Crucially, I argue, Howson’s unique personal and artistic response to the war in Bosnia enables him to maintain not only that trauma is the *appropriate* response to suffering but also that it is the *only* basis for empathy.

A few caveats are necessary. All the official war artists discussed in this article are British. There is nothing universal about their beings-in-the-world. At the most basic, the arguments in this article assume an outsider status for the artist, whose non-traumatised ‘home-self’ moves towards an armed conflict and back again—a luxury not open to most non-Western artists. Similarly, Howson is not ‘representative’ of anything. Nevertheless, his artistic compositions point to an ethically sustainable response to the dilemmas of being an official war artist immersed in a conflict ravaged by genocidal rape. The aim of my article is simply to explore what happens when we think *with* ideas of embodiment and empathy in war art. It will do this through addressing three themes: affective performativity, trauma, and empathy. As I will argue, these three themes are interrelated. In ‘messy’ social worlds, meanings, history, learning, and expectations all influence ways of witnessing war. This is why I conclude with reflections on embodiment and sympathy. * * * 

My term ‘affective performativity’ draws from three theoretical sources: first, the work of Louis Althusser (for the idea of interpellation or the role of ideology as ‘hailing’ the artist into a racialised, gendered, sexualised, and socially classed subject position);^9^ second, the writings of Judith Butler, who argued that performativity is an identity that is always a ‘doing’ or a ‘becoming’, not an innate ‘being’;^10^ and, finally, affect theory, which introduces the embodiment of emotions.^11^

The artist in times of war *does* the identity as ‘war artist’ in negotiation with emotional, bodily, cognitive, and social worlds. At the very basic level, it matters whether their imaginative visions, bodily movements, cognitive processes, and access to material objects (paints and canvas, for example) are categorised as ‘art’ or not. Artists are initiated into aesthetic cultures, from which they make choices. Examples include Howson’s admiration for Pieter Brueghel, Francisco Goya, Otto Dix, and Max Beckmann^12^ compared with David Rowlands’ fascination with the more traditional battlefield artists of the nineteenth century. These entail very different choices: while Howson’s art (as we shall see) is the art of trauma, Rowlands’ art is militaristic and heroic.

As the artist matures, family, friends, acquaintances, reviewers, agents, collectors, and gallery audiences and owners pay attention to some works and not others. These decisions are fundamentally ones of power. It makes a difference if the artist is a working-class, Scottish, white male (Howson) or an English, white woman from a distinguished military and political family (Kitson). Nevertheless, and irrespective of the many ways in which a particular artist is interpellated (or ‘hailed’), bodily comportment and emotional management need to be embedded or embodied—to become ‘second nature’—in the ‘doing’ of the ‘artist’.

These modes of ‘affective performativity’ are highly regulated for artists embedded within the armed forces. Embedded artists are tied to military structures, routines, and assignments. The nature of modern armed conflicts (with their insurgent antagonists, IEDs, and treacherous terrains) means that it is extremely difficult to approach war zones *without* being embedded. As artists have noted, embedded journalists and artists almost inevitably end up identifying strongly with members of their military unit, on whom their lives and livelihoods depend.^13^ Their every sensory faculty becomes profoundly attuned to the hardships suffered by their comrades; the terrors facing civilians or enemy combatants are muted by comparison.

The pressure is not only exerted from the military. The institutions that commission the war artist (in Howson’s case, *The Times* newspaper and the IWM) also have strong views about what they are paying for. Peter Stothard, *The Times*’ editor, expected Howson to ‘reinforc[e]… *The Times*’ commitment to the arts and add… invaluably to *The Times*’ coverage of the war’.^14^ This bureaucratic, pragmatic requirement was accompanied by an aesthetic one: Howson was to acknowledge not only war’s traumas but ‘the heroism and dignity too’.^15^ Commissioned war artists are expected to *perform the emotions* for audiences ‘back home’, encouraging—through a process of contagion—the adoption of those emotions by civilians. Stothard and Howson agreed on the importance of emotional transmission: Stothard lauded the artist’s ‘power to move’,^16^ and prior to deployment Howson admitted that the ‘crunch’ was ‘to see if you can produce work with the ability to move people’.^17^ This bodily metaphor of ‘moving’ is important—as this article argues later when it defines ‘empathy’ as a capacity of both imaginative *and* active (‘moving’ or ‘turning towards’) embodiment.

Nevertheless, the war artists’ ‘set-apartness’ remains crucial for their performance as artists capable of emotionally ‘moving’ people. Howson was well aware of the need to maintain this tension between embeddedness and separateness. He noticed that, as a civilian, he ‘reacted really badly’ the first time he ‘came into contact with something horrible’, whereas ‘all the soldiers had seen it before and, of necessity, could distance themselves from it’. He admitted that ‘Perhaps the same would have happened to me had I spent more time there’, but ‘if it had, perhaps my ability to function as an artist would have been diminished’.^18^

Official war artists have grappled with this tension before. Intrinsic to affective performativity as ‘war artist’ is the disjuncture between home-front rhetoric and combat aesthetics. The home-front rhetoric is exemplified by comments made by *The Times* critic Alan Jackson and Stothard. Jackson praised the IWM and *The Times* for appointing Howson. He contended that Howson’s ‘ability to invest very ordinary men and women with something approaching heroic dignity … makes him an ideal candidate to chronicle an all-too-human war zone’.^19^ Stothard similarly maintained that Howson was the ‘obvious choice to chronicle the catastrophe in Bosnia’ because of his ‘ability to invest ordinary men and women with heroic dignity’.^20^

These comments draw attention to the problem in a stark fashion: after all, Howson’s Bosnian art is anything but ‘heroic’. Indeed, Stothard’s comment was made in a book that included, amongst many other anti-heroic oil paintings, one entitled *Croatian and Muslim* (1994) (fig. 1). Nothing could be further from ‘heroic dignity’ than this scene of sexual assault. Two men press a woman’s head into a toilet while one brutally rapes her. It is a domestic scene, as one of the attackers steadies himself against a family portrait. In the doorway, someone watches. The painting is a repudiation of that distinction between home-front rhetoric and combat aesthetics: the raped woman is at home—a home that is worlds away from those of Jackson and Stothard. The war artist’s affective performativity—his agitated brush marks, thick scrapings of pigment, and frenzied jabs—ultimately *fails* in its contagious function. People in those other, safer ‘homes’ look in horror at the images of rape and carnage but are not ‘moved’ to do anything except gape in shock and awe.

**Figure 1.**
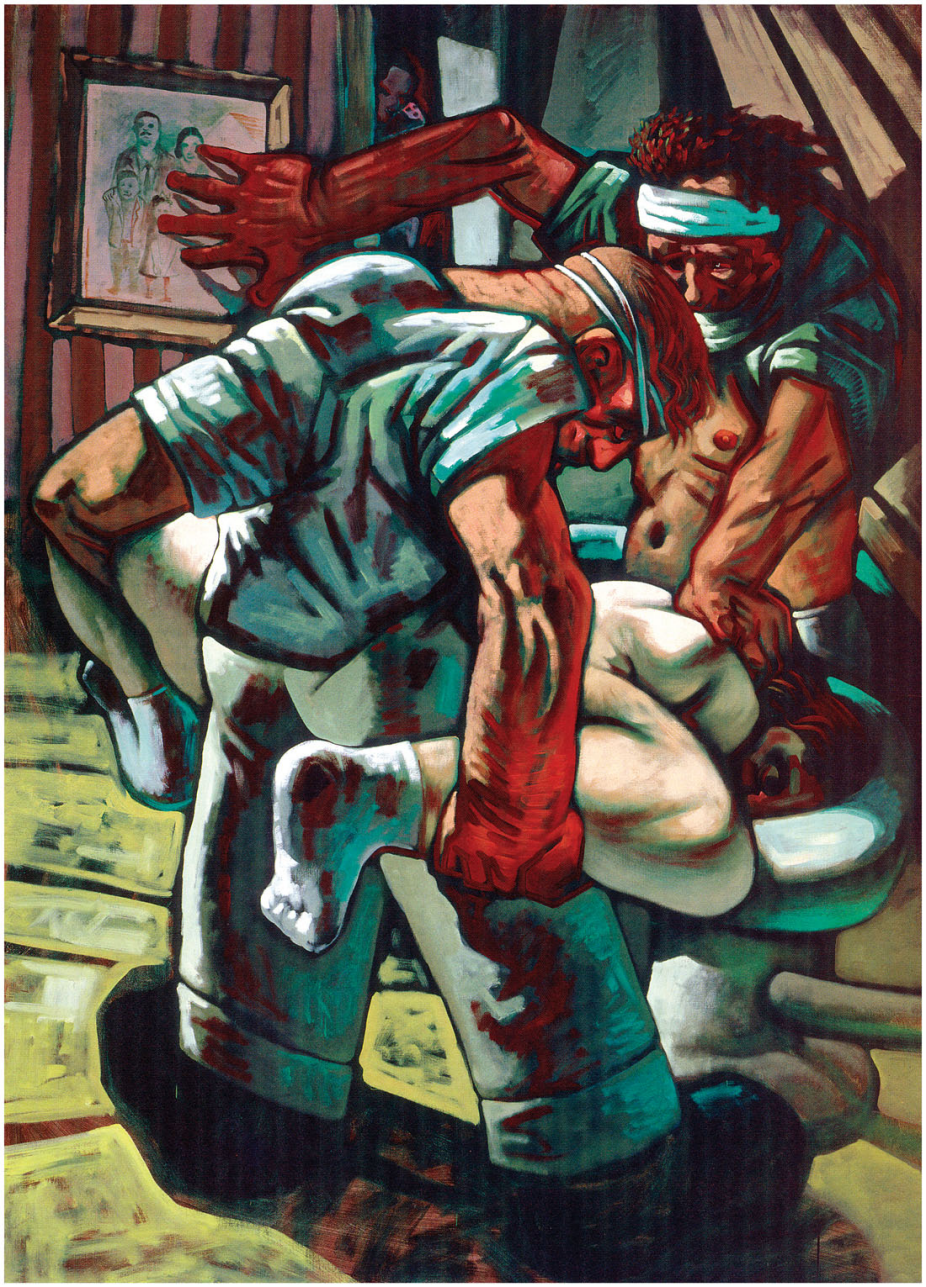
Peter Howson, *Croatian and Muslim*, 1994, oil on canvas, 213 × 152.5 cm. Flowers East, London, where acquired by David Bowie, 6 October 1995. Courtesy of the artist.

If the first tension is the disjuncture between home-front rhetoric and combat aesthetics, the second is between sensory engagement with the armed forces and immersion in battle. In the conventional reception of war art, status adheres to frontline, combat-exposed immersion.

Official war artists can embody three levels. The first is ‘behind the front lines’. Despite the fact that one of the most eminent British war artists of the nineteenth century was a woman—Elizabeth Thompson (Lady Butler)—it remains the case that *official* war artists inhabit masculine personas. The first British woman to occupy the post—Linda Kitson—never got close to the fighting. The worst thing she experienced was ‘extreme weather conditions’.^21^ Because of her gender, she wasn’t even allowed to travel to East Falkland on a Royal Naval vessel. Her appointment gestured towards female equality but actually reinforced sexism. Even Kitson remarked that ‘I think that when there is a girl [sic] about they [servicemen] are very protective. I don’t want to become trite but they do become chivalrous and look after you’.^22^ She admitted that, in getting the commission, it helped that she ‘had the right accent’ and had been born into a distinguished military family.^23^ A disproportionate amount of attention was paid to her clothes: Kitson was described in the press as a ‘small gamin figure in punk-style clothes’, and a large section of her published account of the war was devoted to what she wore. In a foreword to Kitson’s *Visual Diary*, published by the Imperial War Museum, Commander Dennis White patronisingly maintains that ‘It was a privilege to give a little help to a brave, talented and very determined young lady.’^24^ Kitson’s art focuses on everyday activities, disproportionately emphasising senior officers. Only two of ninety images exhibited at the Imperial War Museum include the wounded; the dead have no presence whatsoever.^25^ Even when weapons are depicted, they are either being used in training exercises (rather than combat) or they are merely ‘pictorial motifs’.^26^

At the second level are artists such as John Keane, who was appointed to the post of official ‘war recorder’ during the Gulf War. Like Kitson, he arrived late and had limited exposure to actual fighting. He was embedded on a ship when the violence took the form of aerial bombardment or, for 100 h, fighting on the ground. He ended up being dependent upon his own and *BBC Newsnight*’s footage and photographs.^27^

Keane’s paintings evoke other emotions, however, particularly fear. In his self-portrait *Ecstasy of Fumbling* (1991) (fig. 2), in which he wears a ‘Noddy suit’ (or protective gear) during a suspected gas attack, he looks terrified and confused. Pages torn from *Survive to Fight* are in the background, and in the bottom left-hand corner are a packet of nerve agent pre-treatment tablets and a detection paper for dangerous substances. The title of his painting—a reference to Wilfred Owen’s poem ‘Dulce et Decorum est’—and the postcard of John Singer Sargent’s famous 1919 painting *Gassed* in the lower right-hand corner subtly claim Keane’s status at the heart of an authentic, masculine, and very ‘English’ war culture.

**Figure 2.**
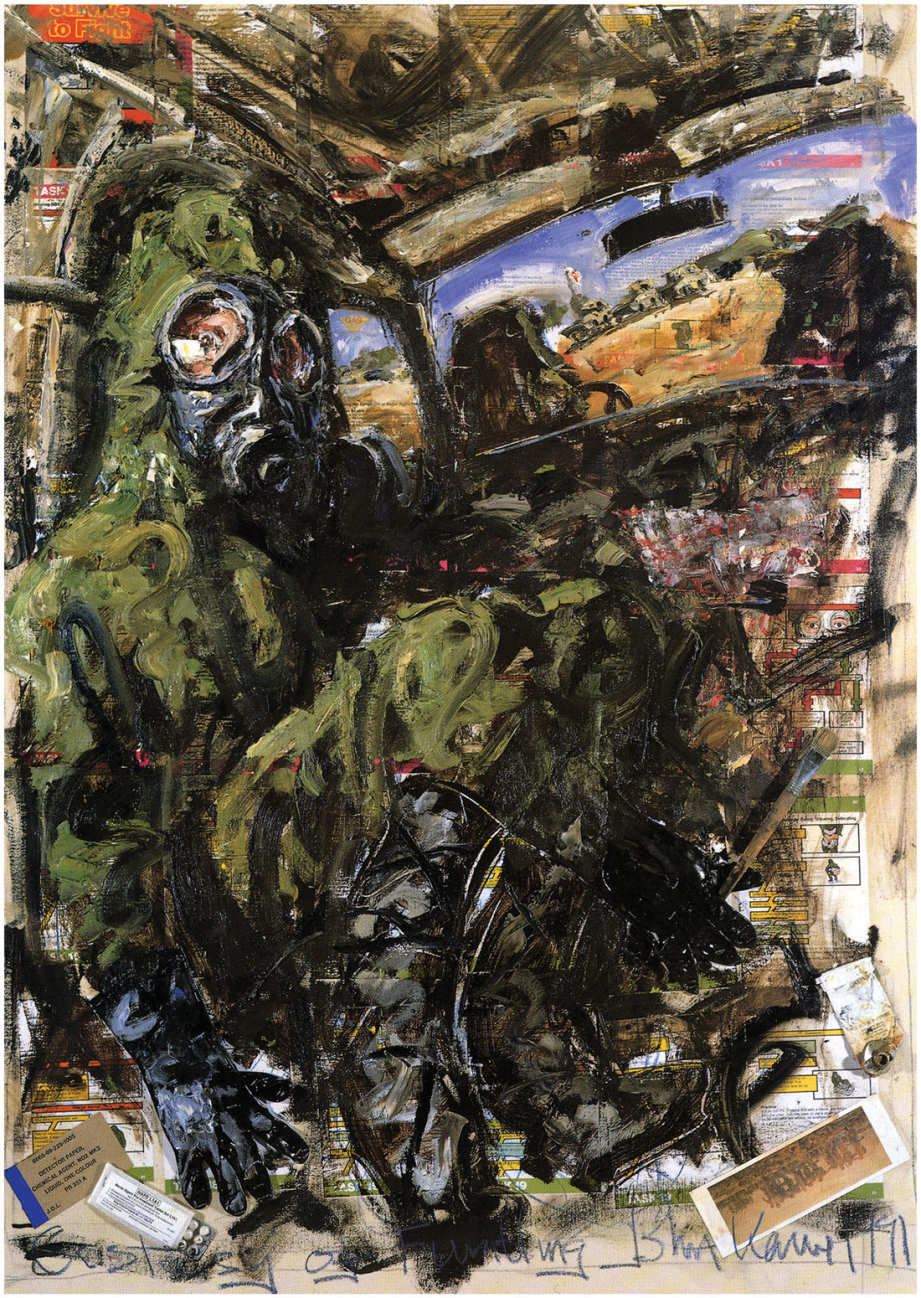
John Keane, *Ecstasy of Fumbling*, 1991, oil and mixed media on canvas, 152 × 107 cm. Courtesy of the artist.

The highest level of authenticity involves male artists who are embedded with combat troops. Howson comported himself quite naturally as an authentic British combatant: he deliberately mimicked soldiers by wearing a uniform, having a ‘No. 1′ hair crop, chain-smoking, and never being seen without a hipflask filled with Scotch.^28^ His exposure to ‘particularly intense’ fighting, including ‘constant sniping and shelling’, was always foregrounded in his account of his time in Bosnia,^29^ as were his encounters with death. In one account, for example, he described a scene of ‘brains and the intestines, studded with fragments of bone and shrapnel’ being scraped off the ground with a shovel and ‘the flies and the terrible, terrible smell’.^30^ Howson believed that he had a ‘right’, as he put it, to do ‘very very frank’ paintings, ‘because I was there and because as an artist, I can do anything’.^31^ Admittedly, his combatant authenticity took a direct hit when he became ill and had to return to his home in Scotland earlier than originally planned.^32^ However, his subsequent return to the front redeemed his reputation. The artist as ‘witness’ to frontline experiences was what gave his paintings authority.

This insistence on ‘authenticity’ is problematic: it involves a masculinist valorisation of violence (both perpetrated and endured) as what ‘maketh the man’. However, there are interesting comparisons to be made between the ‘authenticity’ debates surrounding both Keane’s art and that of Howson. Keane’s oil painting *Mickey Mouse at the Front* (1991) (fig. 3) generated an uproar. The painting shows a barricaded seafront in Kuwait, with dying palm trees (symbolising environmental catastrophe), a shopping trolley full of anti-aircraft rockets (aggressive American consumerism), a crushed Kuwaiti flag, and what many commentators (wrongly) described as ‘a grinning Mickey Mouse squatting upon a plinth as if defecating, an image of America’.^33^ Keane was publicly rebuked for producing ‘inauthentic’ art that was both anti-war and anti-American.

**Figure 3.**
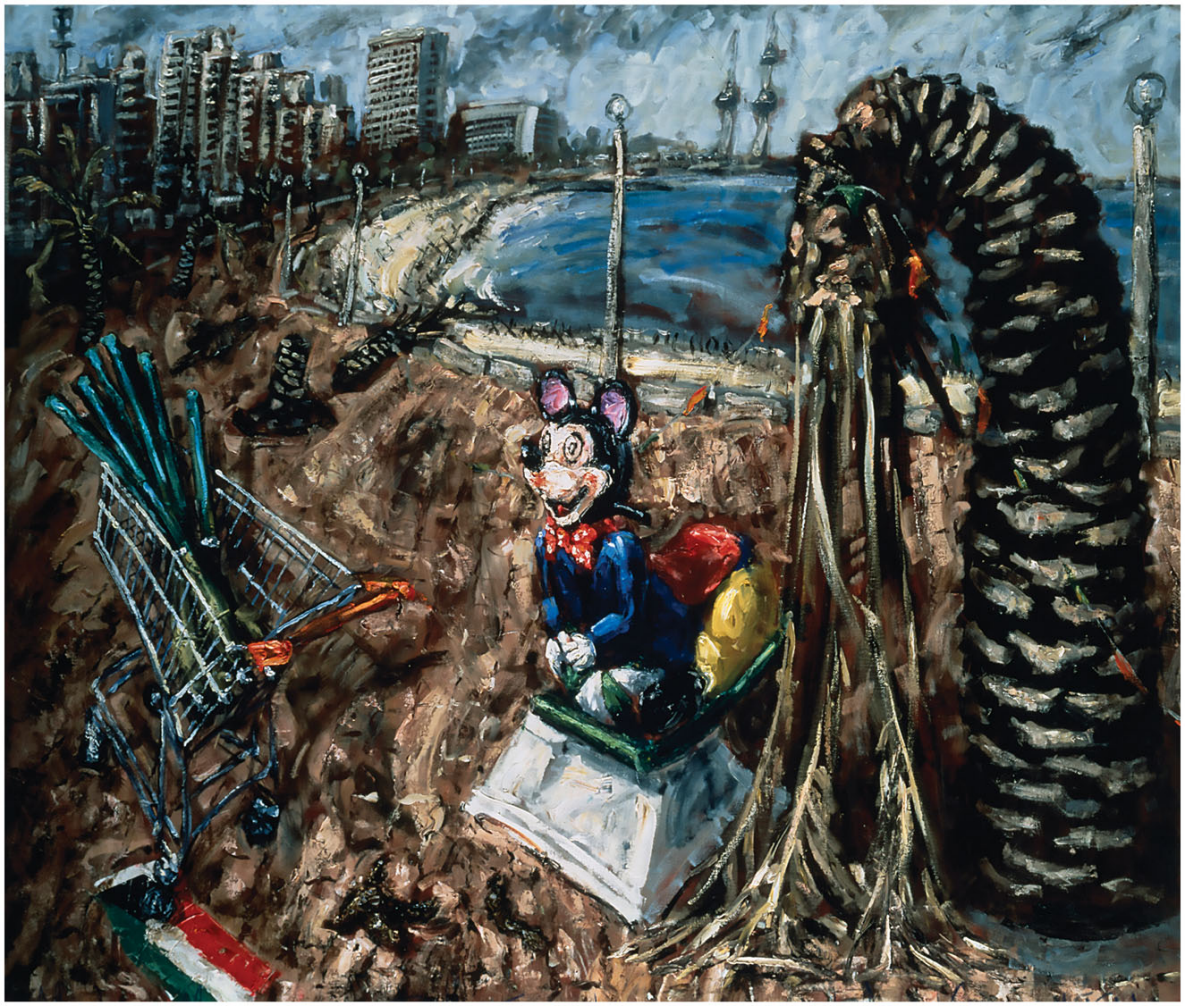
John Keane, *Mickey Mouse at the Front*, 1991, oil on canvas, 173 × 198 cm. Courtesy of the artist.

In contrast, Howson’s *Croatian and Muslim* was castigated for being ‘inauthentic’ for very different reasons. When Howson’s Bosnian paintings were exhibited at the IWM and Flowers East in September 1994, there was an uproar when it was revealed that the IWM, which had a contract with Howson specifying that they could choose works to the value of £20,000 for inclusion in their permanent collection, decided *against* purchasing *Croatian and Muslim*. Instead, they chose *Cleansed*, about Muslim refugees. But two of the five artistic record committee members (IWM’s curator Angela Weight and art critic Marina Vaisey) had preferred *Croatian and Muslim*. The three male committee members (including the banker Jonathan Scott and former Arts Council chairman Sir Kenneth Robinson) had overruled them. IWM’s director-general Alan Borg defended their decision by arguing that *Croatian and Muslim* was inauthentic because Howson had not witnessed the rape firsthand. ‘Although “Croatian and Muslim” is a very strong painting,’ Borg argued, ‘it is a work that could have been produced by any artist sitting in his studio.’^34^ Naturally, this angered Howson. After all, he told Borg,

half of the collection in the Imperial War Museum consists of scenes not actually seen by the artist … The reason why artists are chosen to go to the war is to use their imagination, otherwise they could just send a photographer.^35^

Howson reminded his detractors that Picasso painted *Guernica*, the ‘most famous war painting … without seeing the events’, adding that, although he had not witnessed some of the scenes in his paintings, ‘I could not have done them without going to Bosnia’.^36^ Howson thought that the IWM ‘might have been prompted by the criticism of their choice of a controversial painting by John Keane of the Gulf War’.^37^

The very different critiques of Keane’s and Howson’s paintings are revealing. Keane’s ‘inauthenticity’ lay in his insertion of Mickey Mouse into the war carnage: he was therefore castigated for being anti-war. In contrast, Howson’s inclusion of a *rape* scene was considered ‘inauthentic’ because he was not ‘present’ during the rape of any of the 12,000 to 50,000 estimated victims. In other words, anti-Americanism was considered to be evidence of an anti-war stance; depicting the horrors of wartime rape was not. Rape was naturalised as an inevitable part of war. * * *

If the first theme of this article is affective performativity, the second theme is trauma. The confrontation with violence, and especially its extreme manifestations, hurls witnesses into crisis. Historians of war trauma have explored the *variable* and *embodied* ways that people respond to ‘bad events’ (a term I use advisedly, in order to avoid the historically and culturally specific term ‘trauma’).^38^ A particularly rich historiography exists, tracing changes in the normative, affective performativity of people in the cauldron of combat: this includes shell shock and neurasthenia during the First World War followed by battle exhaustion then post-traumatic stress disorder during the Second World War and the conflict in Vietnam.^39^

Since the 1914–18 war in particular, official war artists have attempted to represent the emotional, bodily, cognitive, and social worlds of trauma in all its historically specific forms. This is not to say that all (or even most) war artists have depicted the horrors of war; they patently haven’t.^40^ It is, however, to suggest that the power of the First World War ‘myth of war’ has had a major effect on subsequent war art. This myth is best exemplified by historian Samuel Hynes’ characterisation of the 1914–18 myth as ‘innocent young men, their heads full of high abstractions like Honour, Glory, and England’ marching off to war in 1914 and becoming disillusioned.^41^ This myth has been especially powerful for commentators who have seen themselves as artist-messengers—here I am thinking of artists such as Paul Nash, who promised ‘bitter truth, and may it burn their lousy souls’.^42^

This First World War–inspired, acrid kind of art is a visual narrative that has proved influential. For war artists keen on representing the ‘authentic’ combat experience, it has become necessary to assault the senses of sight, smell, hearing, taste, and touch of patrons and audiences. Ana Carden-Coyne, David Morris, and Tim Wilcox expanded on this dynamic in their exhibition and book *The Sensory War*. This kind of war art has required artists to visually represent the sound of grenades detonating, the stench of high explosives, the metallic taste of blood, and the sight of human bone, muscle, tissue, skin, hair, and fat strewn around. It has required artists such as Howson to listen to the stories of castration and of brutalised children, corrupted by the violence, and to seek to represent such traumas in paintings such as *Plum Grove* (1994) (fig. 4).^43^ That painting depicts children playing next to a castrated, crucified corpse. The image not only visually represents stories Howson had heard about what was being done to prisoners of war but also speaks to his own terror of castration while in Bosnia. He once stayed with a Croatian family near the army base at Vitez. He later remembered that he was

**Figure 4.**
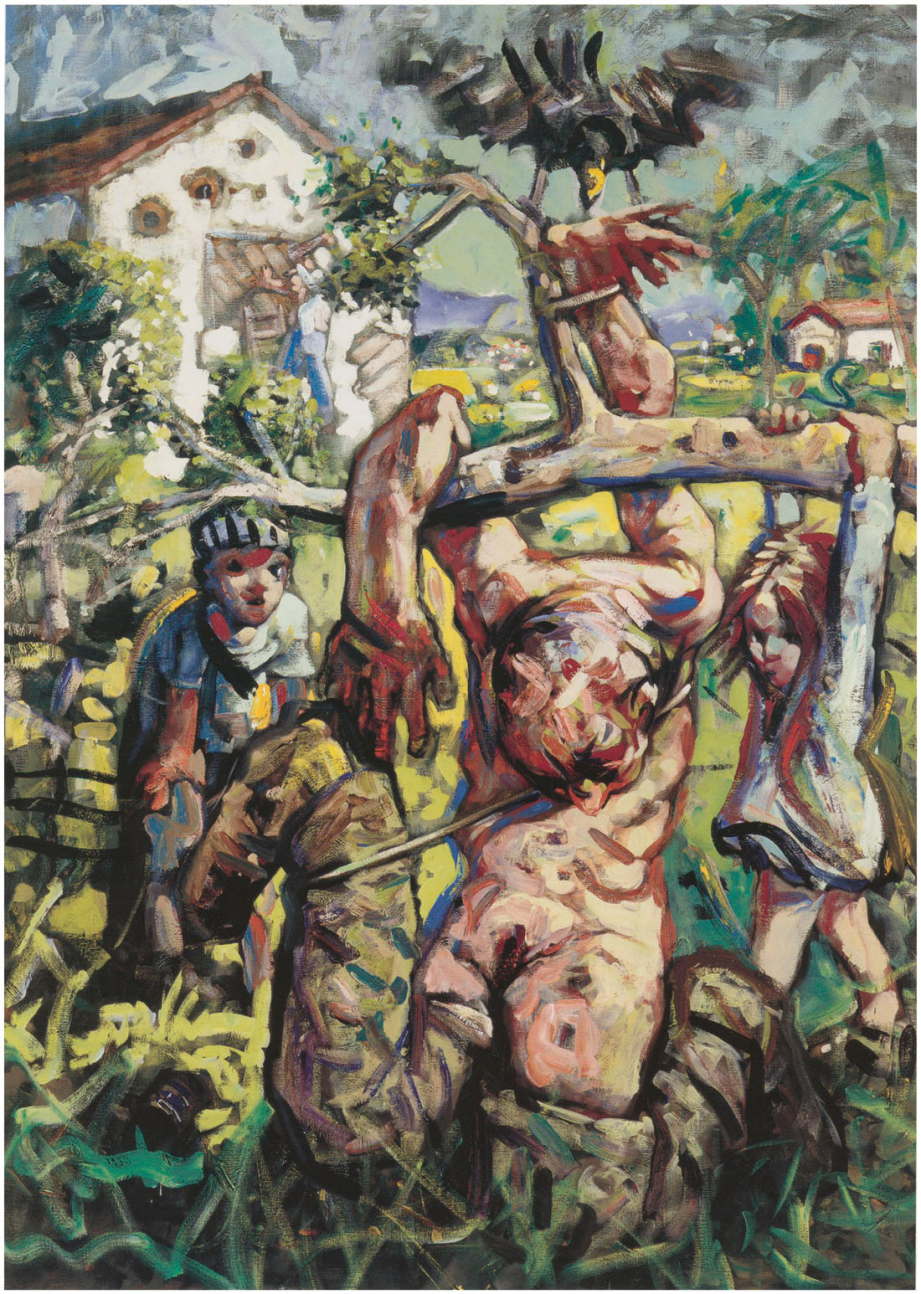
Peter Howson, *Plum Grove*, 1994, oil on canvas, 213.7 × 152.3 cm. Tate. Courtesy of the artist.

lying in bed with my flak jacket and helmet on … I kept imagining the door being kicked open and these guys wearing balaclavas coming in and cutting my bollocks off or kidnapping me. That happens to a lot of people, masked men coming in the middle of the night and killing them or torturing them. It got so bad, I wasn’t sleeping at all.^44^

Like the raped woman, not safe at home, Howson was traumatised by his own utter helplessness. In this mode of war art, artists and their audiences scorn heroics, *insisting* on wounds. To paraphrase Elaine Scarry, ‘to see pain [in war art] is to have certainty—to see heroics is to have doubt’.^45^ In this way, artists perform both the bitterness and the vulnerability of modern war.

However, there have been major shifts in the *ways* that trauma has been portrayed by artists from the First World War onwards. The first of these shifts was the move from the shock of *betrayal* of the body, senses, and mind as a result of encounters with violent death (which was largely the disillusionment motif expressed in the war art of the First World War) to the belief that trauma was *inevitable*, the benchmark of any ‘true’ war experience.^46^ Howson expected—indeed, he planned—to be traumatised. As he later recalled,

This was my opportunity for rebirth, and I was meant to take it. I believe in fate … And I believe 100 per cent that I was meant to go to Bosnia … even though I knew it would be my most traumatic experience ever, and that it would forever change my life and work. I was actually incredibly excited about that aspect of it all. It was a decision I was making for my soul.^47^

He believed that ‘If you don’t get the trauma you don’t get the art … It’s all fear really, the whole thing.’^48^

The second shift relates not to the artist but to their subjects—the victims of atrocity, whose ‘trauma’ is said to be outside of language and other representational modes of expression. Although this is an argument that emerged in the aftermath of the Holocaust, it has become a standard trope of trauma theorists since the 1980s to refer to a wide range of ‘bad experiences’, including woundings, atrocities, ‘disappearances’, and sexual violations.^49^ Unlike independent and experimental artists, *official* war artists are constrained in the mode of representation of trauma. Their job is to provide a visual memory of war for those ‘back home’. This aesthetic can involve making an attempt to communicate suffering.

Of course, this is neither necessary nor required. Many official war artists—even those embedded in combat units—have chosen to ignore or even repudiate the traumas of war, preferring to turn violence into a tempting melodrama or consumable drama. Official war artist David Rowlands is an example. His website is strewn with words and phrases such as ‘glorious deeds’, ‘accurate’, ‘realistic record’, ‘dramatic events of war’, ‘atmospheric’, ‘huge amount of research’, ‘eye-witness participation’, ‘taking part in the patrols and missions’, ‘desolate bravery’, ‘esprit de corps’, and ‘adventures’.^50^

Other official war artists have regarded it as their duty to at least attempt a portrayal of suffering. This struggle of representation is not without pitfalls. In their attempts to represent wartime atrocity, official war artists often proceed by accident; they may stumble in their attempts to communicate with others and often seize upon the nearest, most convenient metaphor. But they recognise that a painful, traumatised world is still a world of meaning. Indeed, the rhetorics of inexpressibility and non-representability can be ways of avoiding ethical engagement. Death and major psychoses are beyond the reach of language and representation, but the vast majority of traumatised people still exist in the world. Trauma is the suffering of survival. * * *

This is where the third theme of this article comes in. My reflections in relation to both affective performativity and trauma have been concerned (in part, at least) with assumptions about the inherent embodiment of consciousness. In this final section, I want to suggest that a kinaesthetic engagement of the senses is central to processes of empathetic identification. The term ‘empathy’ was introduced in 1873 by the German philosopher Robert Vischer. In his book *Über das optische Formgeƒühl: Ein Beitrag zur Aesthetik* (‘On the Optical Sense of Form: A Contribution to Aesthetics’), Vischer describes how, when looking at an object (such as a work of art),

I entrust my individual life to the lifeless form, just as I … do with another living person. Only ostensibly do I remain the same although the object remains an other. I seem merely to adapt and attach myself to it as one hand clasps another, and yet I am mysteriously transplanted and magically transformed into this other.^51^

The viewer, Vischer continues, ‘unconsciously projects its own bodily form—and with this also the soul—into the form of the object. From this I derived the notion that I call “Einƒühlung”’, or empathy.^52^

Vischer’s statement contains the kernel for an understanding of empathy as a capacity of both imaginative *and* active embodiment. Both imagination and action are central to empathetic processes. This is what distinguishes ‘empathy’ from ‘sympathy’: the latter encourages viewers to project their own lived experience of sensation and emotion onto the other person or object (in this case, a painting). ‘Empathy’ does not presume that what is being ‘felt into’ actually *is* what the Other experienced or what the artist intended. It is always at the unattainable edge of imagination; it requires a fully embodied ‘moving’—a ‘moving toward’.^53^

Empathy as a capacity of both imaginative and active embodiment reaches its outermost limit in the face of atrocity. In part, this is because it hurts to see, hear, smell, taste, and touch the vulnerable body of atrocious violence. *The Times* journalist Robert Crampton was half right when he concluded that Howson’s ‘imagination’ was ‘a huge handicap in the struggle to cope’ with the atrocities of war.^54^ Empathy may stall in the face of horror; that stalling may, in fact, be the requirement for survival.

This is where I suggest it is useful to look again at human responses to horror. Many scholars have written about the lure of barbarity. Art historian Suzannah Biernoff, for example, writes about the ‘peculiar power of horror: its power of fascination (for the spectator anyway) and its uncomfortable proximity to pleasure and desire’.^55^ There is an implicit distinction between empathetic aversion to broken, bare life and non-empathetic ensnarement by its horrors. Howson openly struggled with this tension. Acknowledging his attraction to abjection, he reflected that

Half of you detests what you see and half of you wants to be there. You’re living on the edge and it is exciting. That’s the truth of the matter … Someone said the other day, which annoyed me, that the Bosnian work was important and I shouldn’t make it my life’s work, which proved to me that unless you go there you don’t understand how incredible it is. You don’t have a clue … The sixteen days I was there [were] the most intense of my life.^56^

In order to make sense of this paradox—sensory engagement with atrocity as traumatic, but irresistible—there are at least two responses. The first is to return to the pre-modernist idea of ‘mission’. Here, I am not referring to the artist as ‘truth-teller’ (Paul Nash’s promise to tell the ‘bitter truth, and may it burn their lousy souls’) but, rather, as someone whose engagement with the world can show what is unseen by everyone except the victim and their tormentor. I believe that this is what Howson meant in a response to criticisms about his *Croatian and Muslim* painting. He claimed that he did not intend to be ‘controversial’, adding that he was ‘not aiming to do any Mickey Mouse stuff’ (a reference to Keane’s painting). Instead, he explained,

I wanted to cut out all the reportage. It’s not my job to do that. My job is to do the things you don’t see, that the army doesn’t even get to see, not to be an illustrator, not to tell stories, but to produce strong images of things.^57^

Howson’s disavowal of ‘telling stories’ involves a rejection of narrative. The woman whose head is being pushed down the toilet as she is being violated has had her ‘story’ wiped: she has no name, no ‘backstory’. She is nothing—simply ‘Muslim’. *That* is part of her trauma. Howson’s ‘job’ (as he put it) was to show what could not be seen: the rape of a woman who looks askew at us.

The second response conjures up the mimetic version of psychoanalysis, in which trauma involves a compulsive returning to the site of loss: a repetition compulsion or jouissance. This relates to my definition of empathy as a capacity of imaginative *and* active embodiment. Howson openly acknowledged that he was compulsively *compelled* to return to the scene of atrocity after having left it due to illness. This compulsion involved an *active* form of empathetic identification because it required him to re-enter the scene of atrocity in order to help its victims. In a very provocative statement, Howson claimed that it was this face-to-face encounter with mass rape that was ‘one of the greatest days in my life’.^58^ What he meant was not that it was (literally) a wonderful day, but that it was a day that enabled him to take a step towards healing his own trauma of engaged and embodied witnessing. This day was in early December 1993, when he accompanied the British Army as they collected 150 women and children made homeless by the Serbs in the previously Muslim town of Banja Luka. Some had been raped. Howson described what happened next:

When we arrived, we found them cowering in the snow. Gunfire from the surrounding hills was flying over their heads every few seconds, while the Serb soldiers guarding them were very heavily armed and treating them with the most appalling contempt–not to mention doing their level best to taunt and provoke their British counterparts. Iain and I had to make a personal decision about whether to just stand back and observe events unfolding, or whether to leave our position of safety and get actively involved in helping these people, all of them so fearful of what might happen to them that they couldn’t even look you in the eye. Obviously, we got involved, and did our best to help.^59^

Howson’s artistic acts fulfilled a similar function. In *Croatian and Muslim*, Howson was not ‘telling stories’; he was not ‘reporting’ any particular woman’s life experience. He was acknowledging that no-one (let alone an artist) can undo the wound already inflicted. But his art allowed him to ‘work through’ the trauma of witnessing atrocity. The exhibition of this work at the Imperial War Museum enabled his personal melancholic wound to subside, or at least to morph into a more bearable form of mourning. As Howson explained,

I felt totally elated after the opening … For months I’d been troubled by nightmares–awful adventure-dreams, too grotesque to describe–but they stopped immediately. It was as if I’d been able to get the task of Bosnia out of my system at last. Media reports from the war continued to move me, but it was as if my personal responsibility to the conflict had now been fulfilled.^60^

* * * 

In conclusion, the three themes of this article (affective performativity, trauma, and empathy) are interrelated. The responses involved in witnessing suffering do not emerge ‘naturally’ from physiological processes, but in negotiation with ‘messy’ social worlds, including cognitive processes, affective practices, motivations, and even language games. My emphasis has been on the importance of what anthropologist Thomas J. Csordas has called ‘somatic modes of attention’ or ‘culturally elaborated ways of attending to and with one’s body in surroundings that include the embodied presence of others’.^61^ Artists such as Howson do this by their kinaesthetic engagement with paint and brushwork. Howson’s art (and, especially, *Croatian and Muslim*) attempts to bring into being worlds of suffering that had belonged exclusively to victims and their tormentors. At the start of this article, I observed that while it is important not to conflate personal trauma with secondary witnessing, the sights, sounds, smells, tastes, and touch of the wounded body can destroy worlds beyond the immediate victims. *Viewers* of the art of atrocity are being given permission to ‘look’—to stare, even—in ways that would destroy worlds if the suffering-Other was literally in front of them. This argument has been expressed eloquently by photographic theorist Ariella Azoulay, who argues in *Civil Imagination: A Political Ontology of Photography* that viewing a work of war art becomes a civic skill rather than a kind of aesthetic contemplation.^62^ This is not to say that there is an inevitable, inexorable link between keen observation and embodied empathy. Too much evidence suggests exactly the opposite. But by giving permission to stare at the image of terror, such art also gives permission to identify, to empathise—to either look the ‘Muslim’ in the eye or watch voyeuristically from a distance. Our choice.

## Notes

1 This is a reworking of a phrase by Thomas J. Csordas in ‘Somatic Modes of Attention’, *Cultural Anthropology* 8, no. 2 (May 1997): 138.

2 Raymond W.G. Gibbs, ‘Taking Metaphor Out of our Heads and Putting it in the Cultural Worlds’, in *Metaphor in Cognitive Linguistics*, ed. R.W. Gibbs and G.J. Steen (Amsterdam: John Benjamins Publishing Co., 1999), 153.

3 Maurice Merleau-Ponty, ‘The Primacy of Perception’, in *The Primacy of Perception and Other Essays on Phenomenological Psychology, the Philosophy of Art, History, and Politics*, trans. James M. Edie (Evanston: Northwestern University Press, 1964), 42.

4 Maurice Merleau-Ponty, *Phenomenology of Perception* (1945), Part 1 (London: Routledge Classics, 2002).

5 Susan Sontag, *Regarding the Pain of Others* (London: Penguin, 2013) and Susan Sontag, *On Photography* (New York: Picador, 1997).

6 This is a huge literature, but see Dora Apel, *War Culture and the Contest of Images* (New Brunswick: Rutgers University Press, 2012); Jill Bennett, ‘Art, Affect, and the “Bad Death”: Strategies for Communicating the Sense Memory of Loss’, *Signs* 28, no. 1 (Fall 2002), 333–51; Ana Carden-Coyne, David Morris, and Tim Wilcox, eds, *The Sensory War 1914–1918* (Manchester: Manchester Art Gallery, 2014); Cathy Caruth, *Trauma: Explorations in Memory* (Baltimore: John Hopkins University Press, 1995); Cathy Caruth, *Unclaimed Experience: Trauma Narratives and History* (Baltimore: John Hopkins University Press, 2016); Michael Taussig, *Shamanism, Colonialism, and the Wild Man: A Study in Terror and Healing* (Chicago: University of Chicago Press, 1986).

7 For further discussion of the ethics of writing about the art of atrocity, see Joanna Bourke, ed., *War and Art: A Visual History of Modern Conflict* (London: Reaktion Books, 2017).

8 Peter Howson, cited in ‘Artist Aims for Realism’, *The Times*, 30 April 1993, 6.

9 For example, see Louis Althusser, ‘Ideology and the State’, in *Lenin and Philosophy and Other Essays*, trans. B. Brewster (New York: Monthly Review Press, 2001), 85–126.

10 For example, see Judith Butler, *The Psychic Life of Power: Theories in Subjection* (Stanford: Stanford University Press, 1997) and *Excitable Speech: A Politics of the Performative* (New York: Routledge, 1997).

11 For an overview, see Melissa Gregg and Gregory J. Seigworth, *The Affect Theory Reader* (Durham, NC: Duke University Press, 2010). For an insightful critique, see Ruth Leys, *The Ascent of Affect: Genealogy and Critique* (Chicago: University of Chicago Press, 2017).

12 Robert Heller, *Peter Howson* (Edinburgh: Mainstream Publishing, 1993), 17–18.

13 For a critique of embeddedness, see Julian Stallabrass, ‘The Power and Impotence of Images’, in *Memory of Fire*, ed. Julian Stallabrass (Brighton, UK: Brighton Photo Biennial, 2008), http://static.mediapart.fr/files/Stallabrass-Power.pdf.

14 Peter Stothard, ‘Introduction’, in *Peter Howson: Bosnia* (London: Imperial War Museum, 1994), n.p.

15 Ibid.

16 Ibid.

17 Peter Howson, cited in Alan Jackson, ‘The Human Face of War’, *The Times*, 1 May 1993, 9.

18 Peter Howson, cited in Alan Jackson, ‘War at First Hand’, *The Times*, 10 July 1993, 25. Also cited in Alan Jackson, *A Different Man: Peter Howson’s Art, from Bosnia and Beyond* (Edinburgh: Mainstream Publishing, 1997), n.p.

19 Jackson, ‘The Human Face of War’, 9.

20 Stothard, ‘Introduction’, in *Peter Howson: Bosnia*, n.p.

21 Linda Kitson, interview, in Julian Thompson and Linda Kitson, ‘Drawing the Falklands: Julian Thompson and Linda Kitson Discuss War Artists, Art, and Memory’, *The RUSI Journal* 162, no. 2 (2017): 60.

22 Linda Kitson, cited in ‘The Falklands Crisis’, *The Guardian*, 12 May 1982, 2.

23 Kitson, in Thompson and Kitson, ‘Drawing the Falklands’, 61.

24 Commander Dennis White, cited in *The Falklands War: A Visual Diary by Linda Kitson, the Official War Artist* (London: Mitchell Beazley in association with the Imperial War Museum, 1982), n.p.

25 Alex Potts, ‘Eye Witness of the Falklands War’, *History Workshop* 15 (Spring 1983): 193.

26 Ibid., 194.

27 Paul Gough, ‘“Exactitude is Truth”: Representing the British Military Through Commissioned Artworks’, *Journal of War and Culture* 1, no. 3 (2008): 343.

28 Robert Crampton, ‘Facing Fear: Peter Howson in Bosnia’, in *Peter Howson: Bosnia*, n.p.

29 Ibid.

30 Peter Howson, cited in Jackson, *A Different Man*, n.p.

31 Peter Howson, cited in Crampton, ‘Facing Fear’, n.p.

32 Crampton, ‘Facing Fear’, n.p.

33 Julian Stallabrass, ‘Painting Desert Storm’, *New Left Review* (September–October 1992), 103.

34 Alan Borg, cited in Alexandra Frean, ‘Briton’s Bosnian Rape Painting May Go Abroad’, *The Times*, 20 September 1994, 3.

35 Peter Howson, cited in Frean, ‘Briton’s Bosnian Rape Painting May Go Abroad’, 3.

36 Peter Howson, cited in John Young, ‘Painter’s Vision Puts the Horrors of Bosnian Conflict into Perspective’, *The Times*, 22 September 1994, 8.

37 Ibid.

38 For a critique of the uses of the work ‘trauma’ and a defence of the use of the term ‘bad event’, see Joanna Bourke, ‘Sexual Violence, Bodily Pain, and Trauma: A History’, *Theory, Culture and Society* 29, vol. 3 (May 2012), 25–51; Joanna Bourke, ‘Sexual Violence and Trauma in Historical Perspective’, *Arbor* 186 (May–June 2010), 407–16; and Joanna Bourke, *The Story of Pain: From Prayer to Painkillers* (Oxford: Oxford University Press, 2014).

39 This is a large literature, but for representative examples, see Joanna Bourke, *An Intimate History of Killing: Face-to-Face Killing in Twentieth-Century Warfare* (London: Granta, 1999); Carden-Coyne, Morris, and Wilcox, eds, *The Sensory War 1914–1918*; Edgar Jones and Simon Wessley, *Shell Shock to PTSD: Military Psychiatry from 1900 to the Gulf War* (London: Psychology Press, 2005); Peter Leese, *Shell Shock: Traumatic Neurosis and the British Soldier of the First World War* (New York: Palgrave, 2012); Hans Binneveld, *From Shell Shock to Combat Stress: A Comparative History of Military Psychiatry* (Amsterdam: Amsterdam University Press, 1997).

40 For many examples, see Bourke, ed., *War and Art*.

41 Samuel Hynes, *A War Imagined: The First World War and English Culture* (London: Pimlico, 1990), x.

42 Paul Nash to Margaret Nash, 13 November 1917, in Paul Nash, *Outline: An Autobiography and Other Writings* (London: Faber and Faber, 1948), 210–11.

43 This point was also made by Robert Crampton in ‘Facing Fear’, in *Peter Howson: Bosnia*, n.p.

44 Peter Howson, cited in Robert Crampton, ‘To Hell and Back’, *The Times*, 20 November 1993, 32. Also cited in Crampton, ‘Facing Fear’, n.p.

45 Elaine Scarry, *The Body in Pain: The Making and Unmaking of the World* (Oxford: Oxford University Press, 1985), 13.

46 This shift can also be seen in war memoirs: for example, see Joanna Bourke, ‘Bodily Pain, Combat, and the Politics of Memoirs Between the American Civil War and the War in Vietnam’, *Histoire sociale / Social History* 46, no. 91 (May 2013): 43–61 and Joanna Bourke, ‘Pugnacity, Pain, and Professionalism: British Combat Memoirs from Afghanistan, 2007–14’, in *War Stories: The War Memoir in History and Literature*, ed. Philip Dwyer (New York: Berghahn, 2016), 277–301.

47 Howson, cited in Jackson, *A Different Man*, n.p.

48 Peter Howson, cited in Robert Crampton, ‘Blood’, *The Times*, 3 September 1994, 9. Also cited in Crampton, ‘Facing Fear’, n.p.

49 This is a huge literature, but see Caruth, *Trauma*; Caruth, *Unclaimed Experience*; Scarry, *The Body in Pain*. For a critique, see Bourke, *The Story of Pain*.

50 David Rowlands, http://www.davidrowlands.co.uk/index.asp.

51 Robert Vischer, *Über das optische Formgeƒühl: Ein Beitrag zur Aesthetik* (Leipzig: Credner, 1873), 20, in *Empathy, Form, and Space: Problems in German Aesthetics, 1873–1893*, ed. and trans. Harry Francis Mallgrave and Eleftherios Ikonomou (Santa Monica: Getty Center for the History of Art and the Humanities, 1994), 192.

52 Ibid.

53 For further discussion, see Joanna Bourke, ‘Pain, Sympathy, and the Medical Encounter Between the Mid Eighteenth and Mid Twentieth Centuries’, *Historical Research* 85, no. 229 (August 2012): 430–68.

54 Crampton, ‘Facing Fear’, n.p.

55 Suzannah Biernoff, *Portraits of Violence: War and the Aesthetics of Disfigurement* (Ann Arbor: University of Michigan Press, 2017), 6.

56 Howson, cited in Crampton, ‘Facing Fear’, n.p.

57 Howson, cited in Crampton, ‘Blood’, 9.

58 Howson, cited in Jackson, *A Different Man*, 80.

59 Ibid.

60 Ibid., 81.

61 Csordas, ‘Somatic Modes of Attention’, 138.

62 Ariella Azoulay, *Civil Imagination: A Political Ontology of Photography* (London: Verso, 2015).

